# Acute truncal ataxia without nystagmus

**DOI:** 10.1111/ene.16185

**Published:** 2023-12-21

**Authors:** Sergio Carmona, Carlos Martínez, Guillermo Zalazar, Nehzat Koohi, Diego Kaski

**Affiliations:** ^1^ Fundación San Lucas para la Neurociencia Rosario Argentina; ^2^ Hospital Jose Maria Cullen Santa Fe Argentina; ^3^ Hospital de San Luis, Fundación San Lucas para la Neurociencia Rosario Argentina; ^4^ Institute of Neurology University College London London UK; ^5^ Ear Institute University College London London UK

Dear Editor,

We read with interest the paper by Nikles et al. [1], where they present a cohort of patients with stroke‐related dizziness but no nystagmus. We were surprised, however, that no reference was made to our data, published in this same journal, on acute truncal ataxia in patients with acute vertigo without nystagmus [2], which may reflect the timing of their original submission. Despite some methodological differences, our data share similarities.

Nikles et al. [1] retrospectively analysed 961 head and neck magnetic resonance imaging scans and reviewed notes in the 122 confirmed stroke cases to identify vestibular symptoms and ocular motor signs. We explored the frequency of ataxia without concurrent nystagmus in a cross section of patients with acute vertigo who presented to the emergency department at two centres in Argentina (*n* = 71) and the UK (*n* = 24), of whom a total of 30 patients had stroke syndromes [2]. Nikles and colleagues report that nystagmus was present in 50% of their stroke patients. Eighty percent had isolated posterior circulation stroke, and nystagmus was absent in 46% of these patients. In our 30 patients with acute stroke, nystagmus was absent in 40% of patients.

We did not report the nystagmus characteristics in our patients with stroke, but the majority had gaze‐evoked (“direction‐changing”) nystagmus, in keeping with cerebellar involvement [3]. This differs from the findings by Nikles et al., where in all stroke patients with nystagmus this was spontaneous and typically horizontal or "unspecified." In their cohort, 35% of patients with anterior circulation stroke had nystagmus, again all spontaneous. The authors argue that the absence of nystagmus in so many patients with posterior circulation stroke may be because many patients were evaluated by nonspecialists who may have missed subtle abnormalities. This may account for the absence of gaze‐evoked nystagmus in their cohort.

Considering the high number of patients with stroke in whom no nystagmus was observed, the authors recommend the use of the BE‐FAST (balance, eyes, face, arm, speech, time) for evaluation of all patients with acute vertigo, as a number of their patients without nystagmus had facial palsy, dysarthria, and limb ataxia. They further suggest that a more precise examination of ocular motor function (saccades, smooth pursuit, optokinetic nystagmus) may allow detection of subtle signs suggestive of stroke, ideally with video‐oculography. Although we observed abnormal smooth pursuit and hypermetric saccades in five patients with acute truncal ataxia without nystagmus, the effects of age on the ocular motor system make it difficult to ascribe such abnormalities to the acute pathology. Therefore, reliance on the ocular motor assessment may be insufficient to identify central causes of vertigo when nystagmus is absent.

We emphasize the importance of assessing static balance in patients with acute vertigo, particularly those without nystagmus, where moderate or severe acute truncal ataxia predicts a central (vs. inner ear) pathology [4]. Nikles et al. and others have referred to this as acute imbalance syndrome, but we would argue that such a nomenclature does not reflect the cerebellar dysfunction that underpins the cause of the unsteadiness and perhaps therefore degrades its urgency. We propose that acute truncal ataxia without nystagmus is an important subtype of the acute vestibular syndrome.

## AUTHOR CONTRIBUTIONS

**Diego Kaski:** Conceptualization; methodology; formal analysis; writing – original draft; writing – review and editing. **Sergio Carmona:** Conceptualization; methodology; writing – review and editing. **Carlos Martínez:** Conceptualization; writing – review and editing. **Guillermo Zalazar:** Conceptualization; writing – review and editing. **Nehzat Koohi:** Conceptualization; writing – review and editing.

## CONFLICT OF INTEREST STATEMENT

None of the authors has any conflict of interest to disclose.

## Data Availability

The data that support the findings of this study are available from the corresponding author upon reasonable request.
